# Mayday SeaSight: Combined Analysis of Deep Sequencing and Microarray Data

**DOI:** 10.1371/journal.pone.0016345

**Published:** 2011-01-31

**Authors:** Florian Battke, Kay Nieselt

**Affiliations:** Center for Bioinformatics, University of Tübingen, Tübingen, Germany; Ecole Normale Supérieure de Lyon, France

## Abstract

Recently emerged deep sequencing technologies offer new high-throughput methods to quantify gene expression, epigenetic modifications and DNA-protein binding. From a computational point of view, the data is very different from that produced by the already established microarray technology, providing a new perspective on the samples under study and complementing microarray gene expression data. Software offering the integrated analysis of data from different technologies is of growing importance as new data emerge in systems biology studies. Mayday is an extensible platform for visual data exploration and interactive analysis and provides many methods for dissecting complex transcriptome datasets. We present Mayday SeaSight, an extension that allows to integrate data from different platforms such as deep sequencing and microarrays. It offers methods for computing expression values from mapped reads and raw microarray data, background correction and normalization and linking microarray probes to genomic coordinates. It is now possible to use Mayday's wealth of methods to analyze sequencing data and to combine data from different technologies in one analysis.

## Introduction

The ultimate aim of most biological research is to gain an understanding of biological systems and how their constituting parts function together. Traditionally, researchers would focus on a sub-system of interest, e.g. a small regulatory pathway, and try to determine its function. They would conduct a large number of individual experiments, e.g. to quantify the expression level of the genes involved or to measure the abundance of certain metabolites. Today, individual experiments have been partly replaced by high-throughput data generation methods. The age of large-scale high-throughput data generation in biology started with the introduction of microarrays that could be used to measure a large portion of the transcriptome of an organism, cell type, or tissue in parallel. The resulting “*transcriptomics*” data sets necessitated a new kind of analysis software able to efficiently deal with data of that size.

New *deep sequencing* technologies (also called next-generation or second generation sequencing methods) are now available [1]–[3] to study the transcriptome in unprecedented detail (RNA-Seq, [4]). Both, the new techniques and traditional microarrays are successfully being applied to other research areas, such as the study of chromatin immunoprecipitation (ChIP-seq [5] resp. ChIP-chip [6]). Common to these different applications is the enormous size and complexity of the resulting data.

Visual data inspection is often the fastest way to gain insight into these large data sets. Researchers can visually distinguish patterns that automated methods would miss. This process can be the basis for building hypotheses that can then be tested either by applying automated, algorithmic analyses, or by interactively exploring the data. Software for *transcriptomics* analyses must therefore allow to visualize any aspect of the data in a flexible manner and should not impose one single path of analyses. Furthermore, visualizations must be interactive to facilitate data exploration.

A large number of different methods have been developed for automated as well as exploration-driven analysis of complex transcriptomics data. The aim is to reduce data complexity resp. dimensionality and to extract essential information such as regulatory relationships between genes.

Tools for analyzing RNA-Seq data include TopHat [7], which analyzes mapped reads to identify splice junctions between exons. Cufflinks [8], its sister tool, assembles mapped reads into a parsimonious set of transcripts and then estimates the relative abundances of these transcripts based on their supporting reads. CisGenome [9] is an integrated tool for tiling array, ChIP-seq, genome and cis-regulatory element analysis. Commercial packages include ArrayStar (with the QSeq extension), GenomeStudio (Illumina), Partek Genomics Suite, and the CLC bio suite. Only very few tools integrate both microarray and RNA-Seq data and provide a user-friendly interface to many different statistical and data-mining as well as visualization methods. Thorough analyses require the consecutive application of several methods, depending on the nature of the data, the experimental conditions and on observations made during the course of the analysis itself. Tight integration of different analyses methods and statistical tests with the visualizations is thus of utmost importance for efficient analyses.

Mayday [10], [11] is a framework for explorative data analysis. It combines many interactive visualizations with a solid foundation of statistical methods, a data model supporting meta information, classification and data-mining methods and sophisticated filtering and automation approaches into a user-friendly application. While Mayday's initial emphasis was on transcriptomics data, it can also be used to analyze metabolomics, proteomics and many other kinds of numeric data. Mayday requires no background in programming but allows programmers to access its internal data structures either by writing plug-ins or by using its interactive 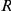 and JavaScript shells.

Here, we present Mayday SeaSight, an extension that allows to integrate sequencing with traditional microarray data and enlarges Mayday's scope of application to a new type of data as well as to integrated analyses of data from different experimental platforms.

Mayday including SeaSight is open-source software available at http://www.microarray-analysis.org.

## Materials and Methods

Mayday [10], [11] is a framework for explorative data analysis. It combines many interactive visualizations with a solid foundation of statistical methods, a data model supporting meta information, classification and data-mining methods and sophisticated filtering and automation approaches into a user-friendly application. Written entirely in Java, it can be installed locally or run without any installation as WebStart application independent of the underlying operating system. Mayday provides efficient core data structures as well as a powerful plugin management system which allows for fast extension via custom plugins. About 80 major plugins are currently included, covering such areas as clustering, filtering, classification, and visualization. Finding significantly differentially expressed genes is another core function covered by Mayday. A host of different statistical methods are already available (e.g. Student's 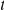-test, SAM [12], Rank Product [13], WAD [14], ANOVA) which can be combined with correction methods for multiple testing.

The fundamental idea underlying Mayday's design is that users should be able to visualize their data in any way they want at any time during the analysis. It offers a range of different visualizations such as scatter plots, box plots, profile plots, enhanced heat maps [15], a genome browser and pathway visualizations [16]. All visualizations are interactive and can be customized in many ways. Many types of meta data (numeric, categorical, statistical, etc.) can be used in visualizations to provide additional information, e.g. by integrating statistical significance into a heat map. All plots can be exported as publication quality files in different formats.

### Microarray, Sequencing and Locus data import

Here we present a new extension for Mayday, SeaSight, that adds a framework for importing raw data from different sources. On the one hand, we have added support for deep sequencing (DS) data. DS methods produce a large number of sequences, called *reads*. A wide range of specialized software packages are available to “map” these reads to a reference genome sequence, i.e. to assign each read to one or more loci within that genome (see [17] for a review). The output of these programs is usually some form of tabular text file. SeaSight offers an import filter for mapped reads that can parse any tabular file as long as the essential information is present. At least, read start positions with respect to genomic coordinates are required. Any further information missing from the file (species, chromosome, read length or end position, strand information) will be requested from the user. SeaSight can also import data stored in the recently introduced mapping file formats SAM and BAM [18]. On the other hand, SeaSight supports importing microarray data from different microarray platforms such as GenePix, Affymetrix, Agilent and ImaGene files, as well as generic tabular files. All imported data is stored in a generic data structure for further processing.

### Working with locus information

When data from microarray and DS experiments are analyzed, or when comparing several DS experiments, a common set of genomic locations must be constructed first. Besides the data import and transformation methods, some of which make use of locus data, SeaSight contains methods for creating, combining, filtering and transforming genetic coordinates. SeaSight can import mappings of identifiers (genes, CDS, ...) to coordinates from files in GenBank, EMBL, Generic Feature (GFF) and Protein Table (PTT) format. Furthermore, we have a very flexible parser for tabular text files supporting any type of column separator, quotation and comment characters and column arrangements. As for the read import step, missing data (species, chromosome, etc.) will be requested from the user. Furthermore, we have a basic algorithm to derive a set of coordinates from DS data based on read counts or sequencing coverage. Locus data can be transformed, for example by changing species or chromosome names using a replacement mapping, by shifting positions, changing feature lengths or strand information, which can be necessary e.g. when using data produced by a non-strand specific protocol.

**Figure 1:**
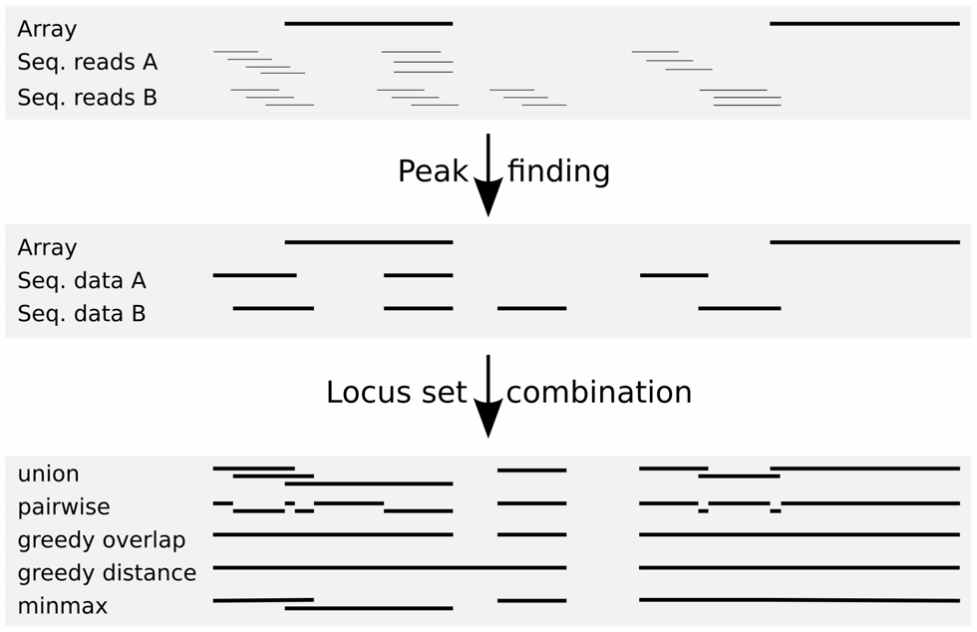
Merging multiple sets of loci. SeaSight offers several methods to combine locus data from different sources. Strong horizontal lines represent genomic loci, fine lines represent reads in the top panel. See text for details.

When several sets of coordinates are present in a data set, they often need to be combined to produce a common set of interrogated positions. We have implemented four methods for this task (see figure 1). The “union” approach uses all unique loci of all input sets, the “pairwise” approach produces one locus for each pair of neighbor coordinates discarding loci not covered by any input set. Both approaches have a minimal size parameter. More sophisticated methods are the “greedy” method which combines loci if they exceed a minimal overlap (or fall below a maximal distance of each other) and the “minmax” method which tries to find maximal extensions by merging overlapping loci within predefined minimal and maximal sizes. Using these methods, users will either get a higher resolution (in terms of genomic coordinates) in the resulting dataset (union, pairwise) or a less sparse expression matrix (greedy, minmax). Depending on the research question, either of these possibilities may be more appropriate.

Finally, SeaSight provides a method to filter one set of coordinates based on another set using a maximal distance, minimal overlap approach.

### Data normalization

**Figure 2:**
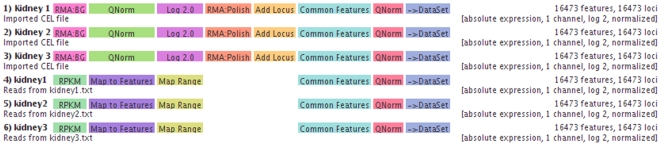
Transformation matrix for part of the case study (using three Affymetrix CEL files and three sequencing result files for the same kidney sample [**21**]). Experiments are displayed as rows, transformations as boxes with color indicating grouping of the transformations. Final data properties are displayed on the right side. Transformations can be added, removed and configured using context menus.

Using data from different sources requires users to carefully decide on a normalization strategy. SeaSight's main element is the *transformation matrix* (see figure 2). Imported data is presented as a list of rows (experiments), which can be freely ordered by the user. Successive transformation steps can be performed on each experiment. These include background correction (subtraction, normexp, RMA), two-channel array normalization (loess, printtip-loess), inter-array normalization (average scaling, percentile scaling, quantile, reference channel quantile), summarization (median polish, mean, median), read count combinations (naive, coverage, RPKM (reads per kilobase exon model per million reads, [19]), DCPM (depth of coverage per base per million reads, [20])), locus-dependent functions (locus import, summarization, mapping), and other transformations (logarithm, interval mapping, MA-transformation, dye-swap, identifier mapping).

Some of these transformations work on single experiments, others work on a (unordered) set of experiments which can be selected using an intuitive interface. This grouping of experiments can be different for each transformation step being added. The goal of these successive steps is to transform each experiment such that all experiments are comparable. The exact definition of “comparable” depends on the respective study. Usually it means that values in different experiments are semantically identical, i.e. a certain numeric value has the same meaning (denotes the same expression strength) regardless of which experiment it appears in.

The successive transformation steps and their input experiments can be represented by a 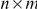 matrix 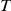, where 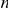 is the number of experiments and 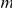 is the total number of successive steps needed to apply all transformations taking into account all opportunities to execute transformations in parallel, i.e. when their input sets of experiments do not overlap. We use the term *transformation instance* for a specific application of a transformation, its input set of experiments and its set of parameters. Then each cell of the matrix contains zero or one transformation instance. Each instance can occupy more than one cell. These cells all occur in the same column but need not be in consecutive rows. Cells can be left empty in which case the data in the respective experiments is not altered in that step. The process of applying the transformations can then be done efficiently by iterating over the columns of the matrix. All transformation instances in a column can be executed in parallel with their respective parameters, using the occupied matrix cells to determine the input experiment set for each instance.

A second matrix 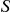 of size 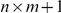 is used internally to model the state of each experiment. The 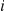
th column contains the state of each experiment before the 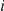
th transformation step. The first column contains the state after parsing, the last column the state after the final transformation is applied. This state matrix is filled before the transformations are applied to the data, at the same time the transformation instances are added to 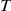. Each transformation method supplies a list of valid input states and the output state after its application to valid input data. These can be used to determine whether a certain transformation is applicable to an experiment at a given transformation step. Thus, SeaSight allows users to quickly add transformation steps to their experiments, without having to wait for lengthy computations to finish. At the same time, a lot of the complexity is hidden from the user. For example, after a background correction was applied to data from a microarray experiment, further background correction is neither possible nor useful.

The resulting state of each experiment is displayed, containing, among others, the number of probes or mapped reads, the number of genetic loci that are present in the experiment, and information on other properties of the data such as the kind of values (absolute vs. relative expression, logged vs. unlogged data, single vs. multichannel array data, etc.).

During the configuration of the transformation matrix, no lengthy calculations are actually performed. Only the state matrix 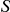 is updated to reflect the data properties resulting from applying the chosen transformations to the data. When satisfied with the predicted result, the user can start the computation to create a dataset for analysis. This dataset can then be stored in Mayday's efficient snapshot file format and shared with other users. Later changes to the transformation matrix are possible to fine-tune the resulting dataset. To this end, the transformation matrix (including all imported data) can be saved to a single, compressed file at any time.

## Results

To illustrate Mayday SeaSight, we use data from a comparative study of RNA-Seq and microarray experiments [21]. Total RNA from liver and kidney samples of a single human male were extracted and each sample was hybridized to three Affymetrix HG-U133 Plus 2.0 microarrays as well as sequenced in three lanes on an Illumina Genome Analyzer, resulting in three technical replicates per platform per tissue. The original paper contains even more replicates and control lanes (see [21] for details) which we did not use in our case study for reasons of clarity. As input for SeaSight, we used the raw microarray data in Affymetrix CEL format and the mapped reads in Eland's (Solexa) output format as available on the original authors' website.

Arrays were normalized using our implementation of the RMA method [22] and locus information for probesets was added from the authors' tabular annotation file. Sequencing data was converted to RPKM values for each locus-annotated feature on the array. The RPKM values were mapped logarithmically to the range 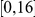 and we used quantile normalization on all samples to create the combined dataset (see figure 2). This dataset, encompassing 12 columns representing the experiments and 16473 rows representing the common features (here transcripts), was then used for subsequent analyses within Mayday.

**Figure 3:**
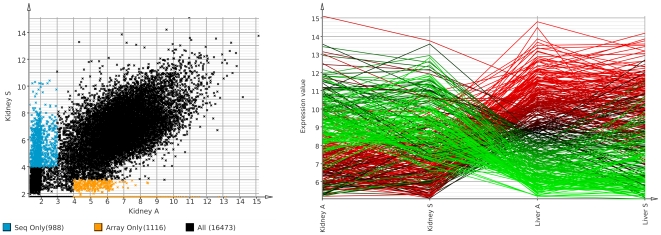
Case study. Total RNA from liver and kidney of a single human male was extracted, sequenced as well as hybridized microarrays. We used three sequencing replicates and three microarray replicates for each tissue. All data processing was done using Mayday SeaSight. Left: Scatter plot of mean replicate gene expression in the kidney sample for array (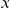 axis) and sequencing data (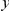 axis) with the genes only found as expressed by one technology highlighted. Right: Visualization of genes reported as differentially expressed between kidney and liver by both sequencing and array data (Rank Product, 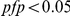). Overall, both technologies show a high agreement. Some genes are only detected by one technology indicating that they complement each other.

To compare the sensitivity of sequencing and array data, we removed genes only found expressed by one technology. First we computed mean expression values from the three technical replicates for each tissue/technology. We call transcripts expressed if their expression value is above 4 and as non-expressed if it is below 3, thus excluding cases where genes would be considered not expressed because they fall just below the expression threshold. Figure 3( left) shows a scatter plot of mean expression values for the kidney sample with the respective sets of genes highlighted. Each technology detects a similar number of genes not found by the other.

Then we removed 6566 genes with low expression values (which are mostly only detected by one of the two technologies) and used Mayday's implementation of the Rank Product method to find differentially expressed genes between the six kidney and the six liver experiments. Genes called as differentially up- resp. downregulated (Rank Product 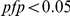) were then visualized in a profile plot (Figure 3 right) using the mean expression for each set of technical replicates and coloring each profile on a red–green gradient depending on its mean expression value in the liver sample array replicates. 156 genes were found to be significantly upregulated in the kidney sample, 176 genes in the liver sample. The next step could now be functional analysis of these genes, for instance using GO terms, or visualizing the most significantly regulated pathways.

Parsing the input files (CEL and CDF files, mapped reads, locus information) and adding all transformations (as shown in figure 2) took five minutes. The computation of the final dataset took less than three minutes. The whole analysis on 30 million mapped reads (970 million bases) and 3.6 million array features was done using Mayday running with 4GB of main memory. All downstream analyses (after the dataset has been constructed) are also possible using Mayday's default of 500 MB memory.

## Discussion

We present SeaSight, a new extension for Mayday, consisting of file parsers for microarray data, mapped sequencing reads and locus information from different sources, as well as a large number of data transformations (platform-specific as well as generic in nature) and operations on locus data. New methods can easily be added as Mayday plugins. Our aim is to provide a user-friendly framework for expression analyses (or more generally transcript-abundance based analyses), in single platform (e.g. Affymetrix microarrays or next-generation sequencing methods), as well as cross-platform scenarios. The intuitive user interface of SeaSight allows to quickly test different normalization strategies while the underlying software design which allows to create “chains” of transformations results in the high flexibility of our approach.

We have implemented processing of large amounts of data in Java, which can be problematic due to the enormous size of non-native datatypes and the overhead inherent in Java collections. To overcome these problems, we implemented our own memory-efficient data structures based on native data types and optimized containers, such as sparse arrays covering genomic regions. These structures scale linearly with the input data size and, in most cases, access to the data is achieved in constant time. We conclude that the widely-held belief that “Java cannot handle large amounts of data” does not apply if programmers take care to design efficient data structures for their particular problem.

Many software packages deal with read mapping, i.e. the assignment of genomic coordinates to each read produced in a deep sequencing experiment. Each algorithm has its own benefits and disadvantages, and the choice of an algorithm and specific parameters depends on the type of experiment. Read mapping is a time-consuming and memory-intensive step that is often done on dedicated computers. Thus we decided to keep mapping and analysis separate and not include a read mapping tool in Mayday. Since all tools provide output in tabular or SAM/BAM format, their output can readily be used with SeaSight.

Although the case study presents only a small portion of Mayday's features, the value of a common importing and processing system such as SeaSight is obvious. Importing raw data and configuring the transformation matrix took only a few minutes, drastically shortening the time researchers have to invest to get their data into a form that allows comparative analyses.

We will continue to develop and implement new methods for Mayday and SeaSight. For instance, the current peak finding algorithm is very basic and was only included as a proof of concept. Until a more sophisticated method is included, we suggest the use of a dedicated peak finding program for this task. Furthermore, the correct choice of normalization methods for DS data as well as for the reconciliation of DS and array data is a field of ongoing research, and also strongly depends on the respective dataset. We do not presume to know the correct method for each case, and the choice of methods employed in the case study is certainly debatable. We think it is important to offer a large number of well-tested methods in one framework, to give researchers a choice to quickly find the right method without having to familiarize themselves with dozens of separate tools.

SeaSight extends Mayday by a powerful data import framework, making Mayday's power as a tool for visual and explorative data analyses available to researchers using new technologies or non-standard experimental pipelines. Furthermore, it facilitates the use of microarray data to validate deep sequencing results. When deep sequencing data is produced without a fully sequenced genome that reads can be mapped to, SeaSight can still be used for analyses given a mapping of identifiers for the measured transcription levels.
